# The Prostate Health Index aids multi-parametric MRI in diagnosing significant prostate cancer

**DOI:** 10.1038/s41598-020-78428-6

**Published:** 2021-03-05

**Authors:** Yu-Hua Fan, Po-Hsun Pan, Wei-Ming Cheng, Hsin-Kai Wang, Shu-Huei Shen, Hsian-Tzu Liu, Hao-Min Cheng, Wei-Ren Chen, Tzu-Hao Huang, Tzu-Chun Wei, I-Shen Huang, Chih-Chieh Lin, Eric Y. H. Huang, Hsiao-Jen Chung, William J. S. Huang, Tzu-Ping Lin

**Affiliations:** 1 Department of Urology, Taipei Veterans General Hospital, No. 201, Section 2, Shipai Road, Taipei, 11217 Taiwan; 2 Department of Urology, School of Medicine, National Yang-Ming University, Taipei, Taiwan; 3 Shu-Tien Urological Institute, National Yang-Ming University, Taipei, Taiwan; 4 Division of Urology, Department of Surgery, Zhongxiao Branch, Taipei City Hospital, Taipei, Taiwan; 5 Department of Radiology, Taipei Veterans General Hospital, Taipei, Taiwan; 6 Center for Evidence-Based Medicine, Taipei Veterans General Hospital, Taipei, Taiwan; 7 School of Medicine, National Yang-Ming University, Taipei, Taiwan; 8 Faculty of Medicine, National Yang-Ming University, Taipei, Taiwan; 9 Institute of Public Health, National Yang-Ming University, Taipei, Taiwan

**Keywords:** Biomarkers, Oncology, Urology

## Abstract

To evaluate the performance of the Prostate Health Index (PHI) in magnetic resonance imaging-transrectal ultrasound (MRI-TRUS) fusion prostate biopsy for the detection of clinically significant prostate cancer (csPCa). We prospectively enrolled 164 patients with at least one Prostate Imaging Reporting and Data System version 2 (PI-RADS v2) ≥ 3 lesions who underwent MRI-TRUS fusion prostate biopsy. Of the PSA-derived biomarkers, the PHI had the best performance in predicting csPCa (AUC 0.792, CI 0.707–0.877) in patients with PI-RADS 4/5 lesions. Furthermore, the predictive power of PHI was even higher in the patients with PI-RADS 3 lesions (AUC 0.884, CI 0.792–0.976). To minimize missing csPCa, we used a PHI cutoff of 27 and 7.4% of patients with PI-RADS 4/5 lesions could have avoided a biopsy. At this level, 2.0% of cases with csPCa would have been missed, with sensitivity and NPV rates of 98.0% and 87.5%, respectively. However, the subgroup of PI-RADS 3 was too small to define the optimal PHI cutoff. PHI was the best PSA-derived biomarker to predict csPCa in MRI-TRUS fusion prostate biopsies in men with PI-RADS ≥ 3 lesions, especially for the patients with PI-RADS 3 lesions who gained the most value.

## Introduction

Prostate specific antigen (PSA) is widely used as a serum marker to detect and monitor the progression of prostate cancer (PCa), and it has dramatically increased the rate of early detection and reduced PCa-specific mortality. However, the low specificity of PSA in determining the presence of PCa and suboptimal ability to discriminate between clinically significant and indolent cancer may lead to unnecessary prostate biopsies and overtreatment, especially in men presenting with a total PSA level of < 10 ng/mL^1,2^.

Accordingly, various PSA-derived biomarkers to detect PCa have been developed^2–5^. The Prostate Health Index (PHI), which is defined as [([-2]proPSA /free PSA) × √total PSA], has been shown to be able to better distinguish PCa from benign prostatic disease compared to other PSA derivatives, and thus help avoid unnecessary prostate biopsies^1,6–9^. The PHI has been adopted into the U.S. National Comprehensive Cancer Network guidelines^10,11^.

Major improvements in multiparametric magnetic resonance imaging (mpMRI) of the prostate with interpretation using the Prostate Imaging Reporting and Data System version 2 (PI-RADS v2) have allowed for reliable visualization of potentially significant PCa, which has improved patient selection for biopsy and facilitated direct targeting of lesions during biopsy^12,13^. Grey et al. used mpMRI to predict biopsy outcomes. Receiver operating characteristic (ROC) curve analysis for clinically significant prostate cancer (csPCa) yielded an area under the curve (AUC) of 0.89, and the negative predictive value (NPV) of a PI-RADS score of ≤ 2 was 0.98^14^. However, the positive predictive value (PPV) for csPCa was 0.49, and a few patients with a PI-RADS score of ≤ 2 had csPCa. Therefore, further improvement of the predictive performance are still needed.

Few studies have evaluated the diagnostic accuracy of PHI combined with mpMRI in predicting PCa^11^. Therefore, we aimed to evaluate the performance of the PHI in cognitive MRI-transrectal ultrasound (MRI-TRUS) fusion-targeted prostate biopsy for the detection of csPCa.

## Materials and methods

### Study design

This was a single-center prospective observational study conducted from March 2017 through July 2019 to determine whether the PHI can improve the detection of csPCa in cognitive MRI-TRUS fusion-targeted prostate biopsies. The Ethics Committee of Taipei Veterans General Hospital has approved this study (IRB number: 2014-02-002AC#5). Written consent was obtained from all of the patients. All methods were performed in accordance with the relevant guidelines and regulations.

The inclusion criteria were patients with at least one PI-RADS ≥ 3 lesion on mpMRI who underwent a cognitive MRI-TRUS fusion-targeted biopsy. All patients had PSA ≥ 4 ng/ml and/or a suspicious digital rectal examination with or without a previous negative systematic TRUS prostate biopsy. The exclusion criteria included acute prostatitis or urinary tract infections, a prior history of PCa, the use of 5-α reductase inhibitors within the previous three months, and having undergone transurethral resection of the prostate. The PHI was evaluated before the prostate biopsy using a Beckman Coulter DxI800 Unicel Immunoassay system.

### mpMRI protocol and reporting

mpMRI was performed using a 3.0 T MRI scanner (MR750, GE Medical Systems, Milwaukee, WI, USA) with a body coil for transmission and a four-coil phased-array torso coil for the reception. The MRI protocol conformed to the European Society of Urogenital Radiology guidelines^15^. The mpMRI included standard anatomical T1- and T2-weighted imaging and functional sequences including dynamic contrast-enhanced and diffusion-weighted imaging, which included the calculation of apparent diffusion coefficient maps. The highest b-value for the diffusion-weighted imaging was 2000s/mm^2^.

Two radiologists, one with more than ten years (S.H.S) and the other with more than five years (H.T.L) of experience in urogenital radiology, reviewed the images together and identified suspicious lesions^16^. The locations of the lesions were assigned according to the 39 regions of interests described in PI-RADS version 2^13^.

### Cognitive MRI-TRUS fusion-targeted biopsy

Patients with suspicious lesions (PI-RADS ≥ 3) following mpMRI received a subsequent targeted biopsy. The TRUS-guided biopsy was performed using an Acuson S3000 ultrasound system (Siemens Medical Solutions, Malvern, PA, USA) with an EC9-4 endocavitary transducer under local anesthesia. The cognitive MRI-targeted biopsy was performed under TRUS-guidance in an axial scan. Three needle passes were performed for each target lesion. A systematic 12-core biopsy was subsequently performed after the targeted biopsy. A single interventional radiologist with over ten years of experience of transrectal biopsy did all procedures. Biopsy samples were analyzed and reported by specialist uropathologists according to the 2005 Consensus Conference of the International Society of Urological Pathology (ISUP)^17^. csPCa was defined as a Gleason score ≥ 7^18^.

### Statistical analysis

The PHI was assessed for its ability to add value to mpMRI in predicting csPCa. Basic statistical analyses of the participants’ characteristics were performed using the Mann–Whitney U test for continuous variables and χ^2^ test for categorical variables. AUC values were estimated for the various PSA derivatives and the difference of AUCs was examined by the DeLong test. Using the cutoff value calculated using the Youden index, logistic regression models were used to predict the detection of PCa. All statistical analyses were carried out using SAS software version 9.4 (SAS Institute Inc., Cary, NC, USA), assuming a two-sided test with a 5% level of significance.

## Results

The clinical characteristics of the 164 patients enrolled are shown in Table 1. The majority of the patients (72%) were biopsy-naive. PCa was detected in 57.3% (94/164) and csPCa in 41.5% (68/164) of the patients. Patients with csPCa were less likely to have a previous negative biopsy than those with non-csPCa (14.7% vs. 37.5%, *P* = 0.001). All of the PSA derivatives were significantly different between the csPCa and non-csPCa groups.

**Table 1:** Demographic and clinical characteristics of patients.

	Total (n = 164)	No cancer (n = 70)	Cancer (n = 94)	*P* value	csPCa (n = 68)	Others* (n = 96)	*P* value
Age, years old	67.0 (62.0–71.0)	66.0 (59.0–69.0)	69.0 (63.8–74.0)	< 0.001	69.0 (63.3–74.8)	66.0 (60.5–69.0)	< 0.001
PNB, N (%)	46.0 (28.0)	29.0 (41.4)	17.0 (18.1)	0.001	10.0 (14.7)	36.0 (37.5)	0.001
Abnormal DRE, N (%)	49.0 (32.5)	6.0 (9.4)	43.0 (49.4)	< 0.001	36.0 (52.9)	13.0 (13.5)	< 0.001
Prostate volume, ml	53.2 (41.0–69.4)	64.9 (43.6–80.3)	48.5 (37.1–61.6)	< 0.001	46.8 (36.9–61.8)	56.8 (43.2–77.1)	0.005
PSAD, ng/ml^2^	0.16 (0.10–0.24)	0.13 (0.08–0.18)	0.20 (0.13–0.32)	< 0.001	0.20 (0.13–0.41)	0.14 (0.09–0.21)	< 0.001
tPSA, ng/ml	9.05 (6.12–12.58)	8.39 (4.83–12.40)	9.08 (6.80–13.43)	0.062	9.05 (7.37–15.05)	9.02 (5.17–11.96)	0.020
%fPSA	0.15 (0.11–0.21)	0.18 (0.13–0.24)	0.13 (0.10–0.18)	< 0.001	0.12 (0.10–0.17)	0.17 (0.12–0.23)	0.001
p2PSA, pg/ml	20.23 (14.35–30.95)	16.17 (11.18–23.66)	22.85 (16.18–38.66)	< 0.001	25.74 (16.28–47.08)	16.69 (12.50–24.00)	< 0.001
%p2PSA	1.60 (1.29–2.09)	1.39 (1.05–1.70)	1.79 (1.46–2.22)	< 0.001	1.94 (1.57–2.49)	1.41 (1.11–1.76)	< 0.001
PHI	44.87 (36.05–61.79)	37.70 (29.70–45.12)	54.62 (41.63–75.71)	< 0.001	58.26 (47.46–94.34)	38.92 (31.81–45.38)	< 0.001

All values given as median (IQR) and number (%).

csPCa, clinically significant prostate cancer; PNB, previous negative biopsy; PSA, prostate-specific antigen; PSAD, PSA density; tPSA, total PSA; fPSA, free PSA; %fPSA, fPSA to tPSA ratio; p2PSA, [-2]pro PSA; %p2PSA, p2PSA to fPSA ratio; PHI, Prostate Health Index.

Others*: no cancer + Gleason sum 6 prostate cancer.

### Biopsy results

Detailed biopsy results are shown in Table 2. The prevalence of csPCa for PI-RADS 3 and 4/5 were 28.6% (16/56) and 48.1% (52/108) respectively. The detection rate of csPCa was significantly higher in targeted biopsies (41.5% vs. 29.3%, *p* < 0.001) compared to systematic biopsies. Targeted biopsies detected significantly fewer Gleason 6 PCa than systematic biopsies (12.2% vs 20.1%, *p* < 0.001). If only targeted biopsies had been performed, six cases of PCa would have been missed, only one of which was csPCa.

**Table 2:** Distribution of PI-RADS score detected by systematic biopsy and MRI-targeted biopsy.

PI-RADS	N	Total prostate cancer		Clinically significant prostate cancer		Gleason sum 6 prostate cancer	
		Targeted, N (%)	Systematic, N (%)	Targeted, N (%)	Systematic, N (%)	Targeted, N (%)	Systematic, N (%)
3	56	20 (35.7)	20 (35.7)	16 (28.6)	15 (26.8)	4 (7.1)	5 (8.9)
4	59	26 (44.1)	24 (40.7)	18 (30.5)	10 (16.9)	8 (13.6)	14 (23.7)
5	49	42 (85.7)	37 (75.5)	34 (69.4)	23 (46.9)	8 (16.3)	14 (28.6)
Total	164	88 (53.7)	81 (49.4)	68 (41.5)	48 (29.3)	20 (12.2)	33 (20.1)

PI-RADS, Prostate Imaging–Reporting and Data System; MRI, Magnetic Resonance Imaging.

### Predictive factors for PCa in patients with PI-RADS 4/5 lesions

**Figure 1:**
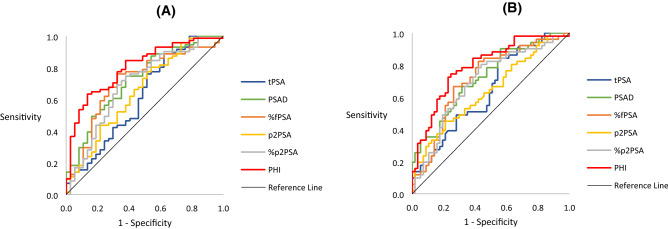
Receiver operating characteristic (ROC) curve analysis for various prostate specific antigen (PSA) derivatives to predict prostate cancer in patients with PI-RADS 4/5. (**A**) Total prostate cancer; (**B**) Clinically significant prostate cancer.

The performance of each PSA derivative in discriminating biopsy outcomes compared with tPSA, as determined by AUC, is presented in Table 3 and Fig. 1. Of all derivatives, the PHI had the best performance in predicting total PCa (AUC 0.802, 95% CI 0.716–0.888) and csPCa (AUC 0.792, 95% CI 0.707–0.877).

**Table 3:** AUC of various PSA derivatives compared to tPSA in patients with PI-RADS 4/5 lesions.

	Total prostate cancer		Clinically significant prostate cancer	
	AUC (95% CI)	*P* value	AUC (95% CI)	*P* value
tPSA	0.617 (0.498–0.736)		0.640 (0.535–0.743)	
PSAD	0.723 (0.621–0.825)	0.0079	0.723 (0.628–0.818)	0.0329
%fPSA	0.713 (0.608–0.818)	0.1778	0.714 (0.615–0.813)	0.2248
p2PSA	0.649 (0.536–0.763)	0.5026	0.637 (0.532–0.742)	0.9690
%p2PSA	0.692 (0.582–0.802)	0.4332	0.694 (0.592–0.795)	0.5055
PHI	0.802 (0.716–0.888)	0.0010	0.792 (0.707–0.877)	0.0027

PI-RADS, Prostate Imaging Reporting and Data System; AUC, area under curve; PSA, prostate-specific antigen; CI, confidence interval; PSAD, PSA density; tPSA, total PSA; fPSA, free PSA; %fPSA, fPSA to tPSA ratio; p2PSA, [-2]pro PSA; %p2PSA, p2PSA to fPSA ratio; PHI, Prostate Health Index.

Using the cutoff value calculated using the Youden index, we performed multivariate logistic regression analysis (Table 4). All the PSA derivatives were significant independent predictors of csPCa at biopsy. PHI had the best performance among the PSA derivatives (*p* < 0.0001). Using a PHI cutoff value of 48.079 for biopsy, 57(52.8%) patients could have avoided undergoing a biopsy, while 27.5% of cases with csPCa would have been missed. To determine the optimal PHI value to minimize missing csPCa, we tested different cutoff values. Using a PHI cutoff value of 27, 7.4% of patients could have avoided a biopsy. At this level, only one (2.0%) case with csPCa would have been missed, with sensitivity and NPV rates of 98.0% and 87.5%, respectively.

**Table 4:** Multivariate logistic regression analyses to predict the detection of clinically significant prostate cancer at biopsy in patients with PI-RADS 4/5 lesions.

Variable	Cutoff	Sensitivity	Specificity	Adjusted OR^a^ (95% CI)	*P* value
tPSA	6.531	0.843	0.439	7.583 (2.093–27.477)	0.0020
%fPSA	0.142	0.667	0.737	0.194 (0.069–0.551)	0.0021
p2PSA	25.280	0.451	0.789	6.545 (1.967–21.774)	0.0022
%p2PSA	1.502	0.804	0.561	4.821 (1.640–14.170)	0.0042
PHI	48.079	0.745	0.754	11.734 (3.437–40.063)	< 0.0001

Excluding PSA density in multivariate model to avoid multi-collinearity problems.

PI-RADS, Prostate Imaging Reporting and Data System; PSA, prostate-specific antigen; tPSA, total PSA; fPSA, free PSA; %fPSA, fPSA to tPSA ratio; p2PSA, [-2]pro PSA; %p2PSA, p2PSA to fPSA ratio; PHI, Prostate Health Index; OR, odds ratio; CI, confidence interval.

^a^Adjusted for age, prostate volume, previous negative biopsy and abnormal digital rectal examination.

### *Diagnostic accuracy of PHI for PCa in patients with PI-RADS 3 lesions (n* = *56)*

**Figure 2:**
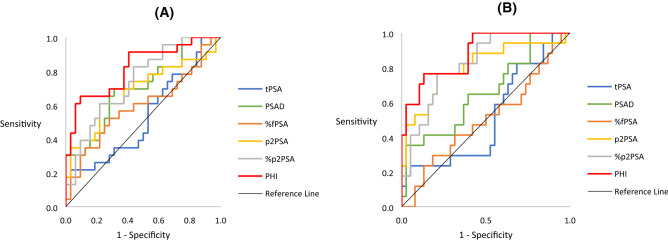
Receiver operating characteristic (ROC) curve for PHI to predict prostate cancer in patients with PI-RADS = 3. (**A**) Total prostate cancer; (**B**) Clinically significant prostate cancer.

As PI-RADS 3 lesions have relatively low rates of csPCa and may represent a new “gray zone” in cognitive MRI-TRUS fusion-targeted prostate biopsy, we performed subgroup analysis of the patients with PI-RADS 3 lesions to investigate whether PHI could improve the predictive ability for PCa compared to tPSA (Table 5 and Fig. 2). Of all derivatives, the PHI had the best performance in predicting total PCa (AUC 0.824, 95% CI 0.710–0.937) and csPCa (AUC 0.884, 95% CI 0.792–0.976). Furthermore, PHI performed better in patients with PI-RADS 3 lesions than that with PI-RADS 4/5 lesions. Using a PHI cutoff value of 50.129 calculated using the Youden index for biopsy, 38(69.1%) patients could have avoided undergoing a biopsy, while 5.9% of cases with csPCa would have been missed. The PHI alone was a significant predictor for csPCa (OR 39.029, 95% CI 2.516–605.358, *P* = 0.0088) after adjusting for age, prostate volume, previous negative biopsy and abnormal digital rectal examination in logistic regression analysis. Nonetheless, the confidence interval was wide which means that the subgroup of PI-RADS 3 was too small to define the optimal cutoff for PHI.

**Table 5:** AUC of various PSA derivatives compared to tPSA in patients with PI-RADS 3 lesions.

	Total prostate cancer		Clinically significant prostate cancer	
	AUC (95% CI)	*P* value	AUC (95% CI)	*P* value
tPSA	0.544 (0.386–0.702)		0.523 (0.352–0.695)	
PSAD	0.711 (0.573–0.850)	0.0022	0.667 (0.507–0.826)	0.0099
%fPSA	0.607 (0.450–0.765)	0.5076	0.516 (0.345–0.687)	0.0931
p2PSA	0.684 (0.531–0.836)	0.1139	0.804 (0.664–0.944)	0.0019
%p2PSA	0.746 (0.617–0.875)	0.0972	0.828 (0.720–0.937)	0.0080
PHI	0.824 (0.710–0.937)	0.0013	0.884 (0.792–0.976)	< 0.0001

PI-RADS, Prostate Imaging Reporting and Data System; AUC, area under curve; PSA, prostate-specific antigen; CI, confidence interval; PSAD, PSA density; tPSA, total PSA; fPSA, free PSA; %fPSA, fPSA to tPSA ratio; p2PSA, [-2]pro PSA; %p2PSA, p2PSA to fPSA ratio; PHI, Prostate Health Index.

## Discussion

Few studies have evaluated the complementary role of PHI and mpMRI in detecting csPCa. To the best of our knowledge, this study is the largest cohort study focusing on the value of PHI in addition to mpMRI in predicting biopsy outcomes in patients with positive mpMRI findings according to PI-RADS version 2 who underwent an MRI-TRUS fusion-targeted biopsy. We found that the PHI was the best PSA-derived biomarker to predict csPCa in a cognitive MRI-TRUS fusion-targeted biopsy in men with PI-RADS 4/5 lesions, with an AUC of 0.792 (CI 0.707–0.877). In addition, an optimal threshold of ≥ 48.079 was defined by the Youden index, at which 27.5% of the cases of csPCa would have been missed and 52.8% of the patients could have avoided a biopsy. Using a PHI cutoff value of 27 to minimize missing csPCa, 7.4% of patients could have avoided a biopsy. At this level, 2.0% of cases with csPCa would have been missed, with sensitivity and NPV rates of 98.0% and 87.5%, respectively.

Gnanapragasam et al. reported the first evidence of the incremental value of adding PHI to mpMRI for the detection of csPCa in patients undergoing a targeted biopsy. In repeat biopsies with a transperineal approach, they found that adding PHI to mpMRI resulted in a much greater improvement in the predictive performance (AUC 0.75, vs. 0.64). Furthermore, using a PHI threshold ≥ 35 among men with negative mpMRI had the highest NPV of 0.97 for excluding csPCa^19^. However, the reporting of mpMRI was based on a Likert scale of cancer probability.

Tosoian et al. reported that none of 15 men with PI-RADS ≤ 3 lesions and PHI < 27 had csPCa on biopsy. In contrast, 29% (8 of 28) of men with PI-RADS ≤ 3 lesions and PHI > 27 had csPCa^20^. However, PHI and mpMRI were performed based on clinical judgment rather than being uniformly obtained in all patients.

In a prospective cohort study recruiting 102 patients with PSA > 4 ng/mL and/or abnormal digital rectal exmaination, Hsieh et al. found that the AUC of combining PHI and mpMRI was 0.873. If biopsies were restricted to patients with PI-RADS ≥ 3 lesions and PHI ≥ 30, 50% of the biopsies could have been avoided with only one csPCa being missed. Of note, patients with negative mpMRI (n = 35) would also have received systematic prostate biopsies in their series^21^.

Actual prevalence of csPCa after a targeted biopsy in PI-RADS 3 lesions varies from 16 to 21%^22^. Even though this prevalence is lower compared to PI-RADS 4–5 lesions, it still reflects that a considerable proportion of men harbor csPCa. As PI-RADS 3 lesions may represent a new “gray zone” in mpMRI, a biomarker is warranted to better stratify higher risk subgroups that harbor csPCa in patients with PI-RADS 3 lesions. This strategy may further help to select patients for targeted biopsy.

Hansen et al. found that a PSAD cutoff value of > 0.15 ng/mL/mL significantly improved the PPV (33%) for csPCa when performing targeted biopsies for PI-RADS 3 lesions^23^. Venderink et al. reported that using a PSAD cutoff value of ≥ 0.15 ng/mL/mL for patients with PI-RADS 3 lesions resulted in 42% of the patients avoiding targeted biopsies, and 6% of cases of csPCa being missed^24^.

PHI has shown promise as a more accurate biomarker than PSAD for predicting csPCa. Unfortunately, few studies have evaluated the incremental value of adding PHI to mpMRI for the detection of csPCa in patients with PI-RADS 3 lesions undergoing targeted biopsies. In the present study, we found that PHI had the highest predictive performance for csPCa with an AUC of 0.884, which was superior to that of PSAD. Nonetheless, the subgroup of PI-RADS 3 was too small (n = 56) to define the optimal cutoff for PHI. Tan et al. reported that a PHI cutoff value of ≥ 27 would have allowed 34% of the patients with PI-RADS 3 lesions (n = 35) to avoid a targeted biopsy, with both sensitivity and NPV of 100%^25^. However, the PHI was evaluated in only half of the patients with a PI-RADS 3 index lesion, which may have resulted in a biased sample.

The value of obtaining mpMRI before a biopsy in biopsy-naive patients is a major topic of interest, as mpMRI is most often recommended in patients with previous negative biopsies^26^. In the present study we only included patients with positive mpMRI, all of whom underwent targeted and systematic biopsies. Overall, 72% of our patients were biopsy-naïve. We found that targeted biopsies detected significantly more csPCa and less clinically insignificant PCa compared to systematic biopsies. If only targeted biopsies were performed, six cases of PCa would have been missed, only one of which was csPCa.

Three prospective multicenter trials have evaluated prebiopsy mpMRI in biopsy-naive patients. The PROMIS trial was designed to assess the utility of mpMRI as a triage test in biopsy-naive patients to avoid unnecessary TRUS-biopsy^27^. The diagnostic accuracy of mpMRI and TRUS-guided biopsy was compared using transperineal template prostate mapping as the reference standard. Ahmed et al. reported that using mpMRI as a triage test resulted in avoiding 27% of primary biopsies, reducing the detection rate of clinically insignificant cancer by 5% and increasing the detection rate of csPCa by 18%. The PRECISION trial then supported the superiority of mpMRI-guided targeted biopsy in biopsy-naive patients^28^. However, the biopsy-naive patients were randomized to receive either a systematic TRUS biopsy without the use of mpMRI, or prebiopsy mpMRI with subsequent biopsy only in patients with positive mpMRI. Therefore, the percentage of the patients with MRI-invisible cancer was unknown. The MRI-FIRST trial offered a prebiopsy mpMRI and both biopsy techniques to all participants rather than randomly assigning them to receive either biopsy technique^29^. Rouviere et al. reported that the omission of a systematic biopsy had no effect on the detection of ISUP grade group ≥ 3 tumors, but that it was associated with a risk of delayed or missed diagnosis for ISUP grade ≤ 2 cancers.

The present study is the largest prospective cohort study to examine the additional value of combining PHI to mpMRI in predicting csPCa in patients with positive mpMRI according to PI-RADS version 2 who underwent MRI-TRUS fusion-targeted biopsies. Furthermore, we highlighted the current controversy regarding the management of PI-RADS 3 lesions, which have relatively low rates of csPCa and may be subject to unnecessary biopsies.

The present study has several limitations. First, the study participants were all drawn from a single tertiary center with an experienced multi-disciplinary diagnostic team, which may limit the generalizability of our findings with regards to primary care patients and the general population. The majority of our patients (72%) were biopsy-naïve. Thus our results may not be applicable to patients with a previous negative biopsy. The subgroup of PI-RADS 3 was too small to define the optimal cutoff for PHI and further efforts are needed to confirm this preliminary finding.

## Conclusions

PHI was the best PSA-derived biomarker to predict csPCa in cognitive MRI-TRUS fusion-targeted biopsy in men with PI-RADS ≥ 3 lesions. Using a PHI cutoff value of ≥ 48.079 defined by Youden index, up to 52.8% of prostate biopsies could have been avoided in patients with PI-RADS 4/5 lesions with missing 27.5% cases of csPCa. In order to minimize missing csPCa, we used a PHI cutoff value of 27 and 7.4% of patients could have avoided a biopsy. At this level, 2.0% of cases with csPCa would have been missed, with sensitivity and NPV rates of 98.0% and 87.5%, respectively.

In patients with PI-RADS 3 index lesions, which is a gray zone for PI-RADS v2, PHI may help identifying high risk groups for csPCa. However, the subgroup of PI-RADS 3 was too small to define the optimal cutoff for PHI. Recruitment of more patients with PI-RADS 3 lesions for optimal cutoff and validation is warranted.
